# Supplementary material for: A 3D basicranial shape-based assessment of local and continental northwest European ancestry among 5th to 9th century CE Anglo-Saxons
Source: PLoS One. 2021 Jun 23;16(6):e0252477. doi: 10.1371/journal.pone.0252477 (PMC8221467; doi:10.1371/journal.pone.0252477)
Supplement: S1 File — (DOCX) [file pone.0252477.s001.docx]

**Supplementary Material for Plomp, Dobney, and Collard’s ‘A 3D**

**basicranial shape-based assessment of local and continental northwest European ancestry among 5^th^ to 9^th^ century CE Anglo-Saxons’**

**Table S1. Locations of the 34 basicranial landmarks used in this study.**

| **Landmark** | **Location** | **Side** |
| --- | --- | --- |
| 1 | Anterior pterion | Left |
| 2 | Anterior pterion | Right |
| 3 | Asterion | Left |
| 4 | Asterion | Right |
| 5 | Lambda | - |
| 6 | Occipital protuberance | - |
| 7 | Parietal notch | Left |
| 8 | Most dorsal end of mastoid notch | Left |
| 9 | Most ventral end of mastoid notch | Left |
| 10 | Mastoidale | Left |
| 11 | Ventral most tip of jugular process | Left |
| 12 | Lateral most point of jugular fossa | Left |
| 13 | Base of styloid process | Left |
| 14 | Medial most point of carotid canal | Left |
| 15 | Ventral most tip of lateral edge of petrous pyramid | Left |
| 16 | Ventral most tip of medial edge of petrous pyramid | Left |
| 17 | Central point of superior border of the auditory meatus | Left |
| 18 | Inferior most point on the entoglenoid process | Left |
| 19 | Ventral most point of lateral border of glenoid fossa | Left |
| 20 | Dorsal most point of lateral border of glenoid fossa | Left |
| 21 | Parietal notch | Right |
| 22 | Most dorsal end of mastoid notch | Right |
| 23 | Most ventral end of mastoid notch | Right |
| 24 | Mastoidale | Right |
| 25 | Ventral most tip of jugular process | Right |
| 26 | Lateral most point of jugular fossa | Right |
| 27 | Base of styloid process | Right |
| 28 | Medial most point of carotid canal | Right |
| 29 | Ventral most tip of lateral edge of petrous pyramid | Right |
| 30 | Ventral most tip of medial edge of petrous pyramid | Right |
| 31 | Central point of superior border of the auditory meatus | Right |
| 32 | Inferior most point on the entoglenoid process | Right |
| 33 | Ventral most point of lateral border of glenoid fossa | Right |
| 34 | Dorsal most point of lateral border of glenoid fossa | Right |

**Table S2. Details of skeletal individuals analysed in this study. Individual = collection number for individual.**

| **Country** | **Institute** | **Site** | **Individual** | **Sex** |
| --- | --- | --- | --- | --- |
| Demark | University of Copenhagen | Lille Vasby | 19 | M |
| Demark | University of Copenhagen | Krongmerken | 12 | F |
| Demark | University of Copenhagen | Simonsborg | S1 | M |
| Demark | University of Copenhagen | Simonsborg | S2 | F |
| Demark | University of Copenhagen | Simonsborg | S5 | F |
| Demark | University of Copenhagen | Simonsborg | S9 | M |
| Demark | University of Copenhagen | Simonsborg | S14 | F |
| Demark | University of Copenhagen | Simonsborg | S31 | M |
| Demark | University of Copenhagen | Varpelev | 5 | F |
| Demark | University of Copenhagen | Varpelev | E | M |
| Demark | University of Copenhagen | Varpelev | J | M |
| Demark | University of Copenhagen | Varpelev | N | M |
| Demark | University of Copenhagen | Vester Egesborg | Ve1 | F |
| Demark | University of Copenhagen | Vester Egesborg | VeA | M |
| Demark | University of Copenhagen | Hesselbjerg | 2 | M |
| Demark | University of Copenhagen | Hesselbjerg | A | M |
| Demark | University of Copenhagen | Hesselbjerg | Gb | M |
| Demark | University of Copenhagen | Hesselbjerg | G5 | M |
| Demark | University of Copenhagen | Hesselbjerg | G6 | M |
| Demark | University of Copenhagen | Hesselbjerg | G30 | M |
| Demark | University of Copenhagen | Hesselbjerg | Ur | M |
| Demark | University of Copenhagen | Hesselbjerg | 4005 | F |
| Demark | University of Copenhagen | Hesselbjerg | 4 | F |
| Demark | University of Copenhagen | Hesselbjerg | Kl | F |
| Demark | University of Copenhagen | Simonsborg | S25 | M |
| Demark | University of Copenhagen | Simonsborg | S26 | M |
| Demark | University of Copenhagen | Simonsborg | S27 | F |
| Demark | University of Copenhagen | Simonsborg | Sh | F |
| Demark | University of Copenhagen | Skyttemarksej | 64 | M |
| Demark | University of Copenhagen | Senderumgaard | Stby | M |
| Demark | University of Copenhagen | Toksvaerd By | Tby | M |
| Demark | University of Copenhagen | Varpelev | V3 | M |
| Demark | University of Copenhagen | Varpelev | V82 | M |
| Demark | University of Copenhagen | Varpelev | 1645 | M |
| Demark | University of Copenhagen | Varpelev | VV1 | M |
| Demark | University of Copenhagen | Smide | 3983 | M |
| Demark | University of Copenhagen | Bakkendrub | 1513 | F |
| Demark | University of Copenhagen | Trelleborg | 1537 | F |
| Demark | University of Copenhagen | Kaagarden | Kel | M |
| Demark | University of Copenhagen | Kaagarden | Kap | M |
| Demark | University of Copenhagen | Kaagarden | Kay | F |
| Demark | University of Copenhagen | Kaagarden | Nbh | M |
| Demark | University of Copenhagen | Kaagarden | Kmlr | M |
| Demark | University of Copenhagen | Kumle Hoje | Khh | M |
| Demark | University of Copenhagen | Treleeborg | Tab | M |
| Demark | University of Copenhagen | Treleeborg | Tsb8 | M |
| Britain | NMNH, London | Poundbury | 15 | M |
| Britain | NMNH, London | Poundbury | 94 | M |
| Britain | NMNH, London | Poundbury | 100 | F |
| Britain | NMNH, London | Poundbury | 107 | M |
| Britain | NMNH, London | Poundbury | 110 | F |
| Britain | NMNH, London | Poundbury | 114 | M |
| Britain | NMNH, London | Poundbury | 118 | M |
| Britain | NMNH, London | Poundbury | 119 | M |
| Britain | NMNH, London | Poundbury | 126 | F |
| Britain | NMNH, London | Poundbury | 142 | M |
| Britain | NMNH, London | Poundbury | 143 | M |
| Britain | NMNH, London | Poundbury | 144 | M |
| Britain | NMNH, London | Poundbury | 155 | F |
| Britain | NMNH, London | Poundbury | 177 | M |
| Britain | NMNH, London | Poundbury | 185 | M |
| Britain | NMNH, London | Poundbury | 207 | M |
| Britain | NMNH, London | Poundbury | 212 | M |
| Britain | NMNH, London | Poundbury | 255 | M |
| Britain | NMNH, London | Poundbury | 276 | F |
| Britain | NMNH, London | Poundbury | 284 | M |
| Britain | NMNH, London | Poundbury | 286 | M |
| Britain | NMNH, London | Poundbury | 290 | M |
| Britain | NMNH, London | Poundbury | 298 | M |
| Britain | NMNH, London | Poundbury | 309 | F |
| Britain | NMNH, London | Poundbury | 322 | F |
| Britain | NMNH, London | Poundbury | 381 | M |
| Britain | NMNH, London | Poundbury | 392 | M |
| Britain | NMNH, London | Poundbury | 393 | M |
| Britain | NMNH, London | Poundbury | 398 | F |
| Britain | NMNH, London | Poundbury | 401 | M |
| Britain | NMNH, London | Poundbury | 403 | M |
| Britain | NMNH, London | Poundbury | 500 | F |
| Britain | NMNH, London | Poundbury | 566 | F |
| Britain | NMNH, London | Poundbury | 568 | F |
| Britain | NMNH, London | Poundbury | 574 | F |
| Britain | NMNH, London | Poundbury | 625 | F |
| Britain | NMNH, London | Poundbury | 638 | F |
| Britain | NMNH, London | Poundbury | 642 | F |
| Britain | NMNH, London | Poundbury | 644 | F |
| Britain | NMNH, London | Poundbury | 650 | M |
| Britain | NMNH, London | Poundbury | 654 | F |
| Britain | NMNH, London | Poundbury | 658 | M |
| Britain | NMNH, London | Poundbury | 679 | F |
| Britain | NMNH, London | Poundbury | 684 | F |
| Britain | NMNH, London | Poundbury | 707 | F |
| Britain | NMNH, London | Poundbury | 1004 | F |
| Britain | NMNH, London | Poundbury | 1022 | F |
| Britain | NMNH, London | Poundbury | 1030 | F |
| Britain | NMNH, London | Poundbury | 1095 | F |
| Britain | NMNH, London | Poundbury | 1225 | M |
| Britain | NMNH, London | Poundbury | 19 | M |
| Britain | NMNH, London | Poundbury | 31 | F |
| Britain | NMNH, London | Poundbury | 103 | F |
| Britain | NMNH, London | Poundbury | 109 | M |
| Britain | NMNH, London | Poundbury | 113 | M |
| Britain | NMNH, London | Poundbury | 121 | F |
| Britain | NMNH, London | Poundbury | 208 | M |
| Britain | NMNH, London | Poundbury | 221 | M |
| Britain | NMNH, London | Poundbury | 247 | M |
| Britain | NMNH, London | Poundbury | 277 | M |
| Britain | NMNH, London | Poundbury | 305 | F |
| Britain | NMNH, London | Poundbury | 385 | F |
| Britain | NMNH, London | Poundbury | 515 | F |
| Britain | NMNH, London | Poundbury | 352 | F |
| Britain | Duckworth Laboratory, University of Cambridge | Hallett’s Garage | 1347 | M |
| Britain | Duckworth Laboratory, University of Cambridge | Hallett’s Garage | 1519 | F |
| Britain | Duckworth Laboratory, University of Cambridge | Hallett’s Garage | 1887 | M |
| Britain | Duckworth Laboratory, University of Cambridge | Hallett’s Garage | 1983 | M |
| Britain | Duckworth Laboratory, University of Cambridge | Hallett’s Garage | 2045 | M |
| Britain | Duckworth Laboratory, University of Cambridge | Maiden Castle | 64 | F |
| Britain | Duckworth Laboratory, University of Cambridge | Maiden Castle | 69 | M |
| Britain | Duckworth Laboratory, University of Cambridge | Maiden Castle | 78 | F |
| Britain | Duckworth Laboratory, University of Cambridge | Maiden Castle | 80 | M |
| Britain | Duckworth Laboratory, University of Cambridge | Maiden Castle | 92 | M |
| Britain | Duckworth Laboratory, University of Cambridge | Maiden Castle | 94 | M |
| Britain | Duckworth Laboratory, University of Cambridge | Maiden Castle | 95 | M |
| Britain | Duckworth Laboratory, University of Cambridge | Maiden Castle | 97 | M |
| Britain | Duckworth Laboratory, University of Cambridge | Maiden Castle | 98 | M |
| Britain | Duckworth Laboratory, University of Cambridge | Maiden Castle | 101 | F |
| Britain | Duckworth Laboratory, University of Cambridge | Maiden Castle | 105 | M |
| Britain | Duckworth Laboratory, University of Cambridge | Maiden Castle | 110 | M |
| Britain | Duckworth Laboratory, University of Cambridge | Maiden Castle | 112 | M |
| Britain | Duckworth Laboratory, University of Cambridge | Maiden Castle | 61 | F |
| Britain | Duckworth Laboratory, University of Cambridge | Maiden Castle | 62 | F |
| Britain | Duckworth Laboratory, University of Cambridge | Maiden Castle | 71 | M |
| Britain | Duckworth Laboratory, University of Cambridge | Maiden Castle | 115 | F |
| Britain | Duckworth Laboratory, University of Cambridge | Maiden Castle | 63 | M |
| Britain | Duckworth Laboratory, University of Cambridge | Maiden Castle | 74 | M |
| Britain | Duckworth Laboratory, University of Cambridge | Maiden Castle | 75 | F |
| Britain | Duckworth Laboratory, University of Cambridge | Maiden Castle | 76 | F |
| Britain | Duckworth Laboratory, University of Cambridge | Maiden Castle | 77 | F |
| Britain | Duckworth Laboratory, University of Cambridge | Maiden Castle | 79 | F |
| Britain | Duckworth Laboratory, University of Cambridge | Maiden Castle | 90 | M |
| Britain | Duckworth Laboratory, University of Cambridge | Maiden Castle | 99 | F |
| Britain | Duckworth Laboratory, University of Cambridge | Maiden Castle | 102 | M |
| Britain | Duckworth Laboratory, University of Cambridge | Maiden Castle | 104 | M |
| Britain | Duckworth Laboratory, University of Cambridge | Maiden Castle | 959 | M |
| Britain | Duckworth Laboratory, University of Cambridge | Maiden Castle | 60 | F |
| Britain | Duckworth Laboratory, University of Cambridge | Maiden Castle | 70 | M |
| Britain | Duckworth Laboratory, University of Cambridge | Maiden Castle | 89 | F |
| Britain | Royal College of Surgeons, London | Breedon-on-the-Hill | 1 | F |
| Britain | Royal College of Surgeons, London | Breedon-on-the-Hill | 9 | F |
| Britain | Royal College of Surgeons, London | Breedon-on-the-Hill | 10 | F |
| Britain | Royal College of Surgeons, London | Breedon-on-the-Hill | 28 | F |
| Britain | Royal College of Surgeons, London | Breedon-on-the-Hill | 68 | F |
| Britain | Royal College of Surgeons, London | Breedon-on-the-Hill | 71 | F |
| Britain | Royal College of Surgeons, London | Breedon-on-the-Hill | 106 | F |
| Britain | Royal College of Surgeons, London | Breedon-on-the-Hill | 117 | F |
| Britain | Royal College of Surgeons, London | Breedon-on-the-Hill | 124 | F |
| Britain | Royal College of Surgeons, London | Breedon-on-the-Hill | 144 | F |
| Britain | Royal College of Surgeons, London | Breedon-on-the-Hill | 188 | F |
| Britain | Royal College of Surgeons, London | Breedon-on-the-Hill | 196 | F |
| Britain | Royal College of Surgeons, London | Breedon-on-the-Hill | 209 | F |
| Britain | Royal College of Surgeons, London | Breedon-on-the-Hill | 3 | M |
| Britain | Royal College of Surgeons, London | Breedon-on-the-Hill | 4 | M |
| Britain | Royal College of Surgeons, London | Breedon-on-the-Hill | 24 | M |
| Britain | Royal College of Surgeons, London | Breedon-on-the-Hill | 97 | M |
| Britain | Royal College of Surgeons, London | Breedon-on-the-Hill | 110 | M |
| Britain | Royal College of Surgeons, London | Breedon-on-the-Hill | 116 | M |
| Britain | Royal College of Surgeons, London | Breedon-on-the-Hill | 118 | M |
| Britain | Royal College of Surgeons, London | Breedon-on-the-Hill | 120 | M |
| Britain | Royal College of Surgeons, London | Breedon-on-the-Hill | 121 | M |
| Britain | Royal College of Surgeons, London | Breedon-on-the-Hill | 122 | M |
| Britain | Royal College of Surgeons, London | Breedon-on-the-Hill | 134 | M |
| Britain | Royal College of Surgeons, London | Breedon-on-the-Hill | 180 | M |
| Britain | Canterbury Archaeological Trust | Dover Buckland | 420 | F |
| Britain | Canterbury Archaeological Trust | Dover Buckland | 425 | F |
| Britain | Canterbury Archaeological Trust | Dover Buckland | 328 | F |
| Britain | Canterbury Archaeological Trust | Dover Buckland | 385 | F |
| Britain | Canterbury Archaeological Trust | Dover Buckland | 327 | F |
| Britain | Canterbury Archaeological Trust | Dover Buckland | 409 | M |
| Britain | Canterbury Archaeological Trust | Dover Buckland | 306 | M |
| Britain | Canterbury Archaeological Trust | Dover Buckland | 290 | M |
| Britain | Suffolk Archaeological Service | Eriswell | 203 | F |
| Britain | Suffolk Archaeological Service | Eriswell | 333 | F |
| Britain | Suffolk Archaeological Service | Eriswell | 457 | F |
| Britain | Suffolk Archaeological Service | Eriswell | 570 | F |
| Britain | Suffolk Archaeological Service | Eriswell | 600 | F |
| Britain | Suffolk Archaeological Service | Eriswell | 612 | F |
| Britain | Suffolk Archaeological Service | Eriswell | 4411 | F |
| Britain | Suffolk Archaeological Service | Eriswell | 2 | M |
| Britain | Suffolk Archaeological Service | Eriswell | 4 | M |
| Britain | Suffolk Archaeological Service | Eriswell | 302 | M |
| Britain | Suffolk Archaeological Service | Eriswell | 384 | M |
| Britain | Suffolk Archaeological Service | Eriswell | 4238 | M |
| Britain | Suffolk Archaeological Service | Brandon | 784 | F |
| Britain | Suffolk Archaeological Service | Brandon | 854 | F |
| Britain | Suffolk Archaeological Service | Brandon | 855 | F |
| Britain | Suffolk Archaeological Service | Brandon | 859 | F |
| Britain | Suffolk Archaeological Service | Brandon | 880 | F |
| Britain | Suffolk Archaeological Service | Brandon | 881 | F |
| Britain | Suffolk Archaeological Service | Brandon | 885 | F |
| Britain | Suffolk Archaeological Service | Brandon | 886 | F |
| Britain | Suffolk Archaeological Service | Brandon | 894 | F |
| Britain | Suffolk Archaeological Service | Brandon | 3056 | F |
| Britain | Suffolk Archaeological Service | Brandon | 3058 | F |
| Britain | Suffolk Archaeological Service | Brandon | 785 | M |
| Britain | Suffolk Archaeological Service | Brandon | 882 | M |
| Britain | Suffolk Archaeological Service | Brandon | 883 | M |
| Britain | Suffolk Archaeological Service | Brandon | 884 | M |
| Britain | Suffolk Archaeological Service | Brandon | 889 | M |
| Britain | Suffolk Archaeological Service | Brandon | 891 | M |
| Britain | Suffolk Archaeological Service | Brandon | 892 | M |
| Britain | Suffolk Archaeological Service | Brandon | 895 | M |
| Britain | Suffolk Archaeological Service | Brandon | 2439 | M |
| Britain | Suffolk Archaeological Service | Brandon | 2954 | M |
| Britain | Suffolk Archaeological Service | Brandon | 3055 | M |
| Britain | Suffolk Archaeological Service | Brandon | 3057 | M |
| Britain | Suffolk Archaeological Service | Brandon | 3060 | M |
| Britain | Suffolk Archaeological Service | Brandon | 3072 | M |
| Britain | Suffolk Archaeological Service | Brandon | 3073 | M |
| Britain | Suffolk Archaeological Service | Burwell | 27 | F |
| Britain | Suffolk Archaeological Service | Burwell | 35 | F |
| Britain | Suffolk Archaeological Service | Burwell | 56 | M |
| Britain | Suffolk Archaeological Service | Burwell | 36 | M |
| Britain | Suffolk Archaeological Service | Burwell | 16 | M |
| Britain | Suffolk Archaeological Service | Burwell | 18 | M |
| Britain | Suffolk Archaeological Service | Burwell | 19 | M |
| Britain | Suffolk Archaeological Service | Burwell | 24 | M |
| Britain | Suffolk Archaeological Service | Burwell | 25 | M |
| Britain | Suffolk Archaeological Service | Burwell | 28 | M |
| Britain | Suffolk Archaeological Service | Burwell | 29 | M |
| Britain | Suffolk Archaeological Service | Burwell | 30 | M |
| Britain | Suffolk Archaeological Service | Burwell | 32 | M |
| Britain | Suffolk Archaeological Service | Burwell | 33 | M |
| Britain | Suffolk Archaeological Service | Burwell | 37 | M |
| Britain | Suffolk Archaeological Service | Burwell | 38 | M |

**Table S3. LDA scores for each Anglo-Saxon female analysed. Individual = collection number for individual.**

| **Population** | **Individual** | **Likelihood Frequency** | |
| --- | --- | --- | --- |
|  |  | Danish | Pre-Medieval British |
| **Early Anglo-Saxons** |  |  |  |
| Breedon-on-the-Hill | 1 | 6% | 94% |
| Breedon-on-the-Hill | 9 | 43% | 54% |
| Breedon-on-the-Hill | 10 | 83% | 17% |
| Breedon-on-the-Hill | 28 | 74% | 26% |
| Breedon-on-the-Hill | 68 | 71% | 29% |
| Breedon-on-the-Hill | 71 | 3% | 97% |
| Breedon-on-the-Hill | 106 | 89% | 11% |
| Breedon-on-the-Hill | 117 | 97% | 3% |
| Breedon-on-the-Hill | 124 | 100% | 0% |
| Breedon-on-the-Hill | 144 | 83% | 17% |
| Breedon-on-the-Hill | 188 | 91% | 9% |
| Breedon-on-the-Hill | 196 | 23% | 77% |
| Breedon-on-the-Hill | 209 | 77% | 23% |
| Dover Buckland | 420 | 26% | 74% |
| Dover Buckland | 425 | 74% | 26% |
| Dover Buckland | 328 | 49% | 51% |
| Dover Buckland | 385 | 100% | 0% |
| Dover Buckland | 327 | 54% | 46% |
| Eriswell | 203 | 31% | 69% |
| Eriswell | 333 | 100% | 0% |
| Eriswell | 457 | 80% | 20% |
| Eriswell | 570 | 80% | 20% |
| Eriswell | 600 | 94% | 6% |
| Eriswell | 612 | 94% | 6% |
| Eriswell | 4411 | 89% | 11% |
| **Middle Anglo-Saxons** |  |  |  |
| Brandon | 784 | 6% | 94% |
| Brandon | 854 | 40% | 60% |
| Brandon | 855 | 69% | 31% |
| Brandon | 859 | 83% | 17% |
| Brandon | 880 | 11% | 89% |
| Brandon | 881 | 23% | 77% |
| Brandon | 885 | 49% | 51% |
| Brandon | 886 | 17% | 83% |
| Brandon | 894 | 26% | 74% |
| Brandon | 3056 | 100% | 0% |
| Brandon | 3058 | 100% | 0% |
| Burwell | 27 | 29% | 71% |
| Burwell | 35 | 69% | 31% |

**Table S4. LDA scores for each Anglo-Saxon male analysed. Individual = collection number for individual.**

| **Population** | **Individual** | **Likelihood Frequency** | |
| --- | --- | --- | --- |
|  |  | Danish | British |
| **Early Anglo-Saxon** |  |  |  |
| Breedon-on-the-Hill | 3 | 96% | 3% |
| Breedon-on-the-Hill | 4 | 100% | 0% |
| Breedon-on-the-Hill | 24 | 43% | 57% |
| Breedon-on-the-Hill | 97 | 50% | 50% |
| Breedon-on-the-Hill | 110 | 21% | 79% |
| Breedon-on-the-Hill | 116 | 39% | 61% |
| Breedon-on-the-Hill | 118 | 100% | 0% |
| Breedon-on-the-Hill | 120 | 71% | 29% |
| Breedon-on-the-Hill | 121 | 96% | 4% |
| Breedon-on-the-Hill | 122 | 100% | 0% |
| Breedon-on-the-Hill | 134 | 39% | 61% |
| Breedon-on-the-Hill | 180 | 89% | 11% |
| Dover Buckland | 409 | 96% | 4% |
| Dover Buckland | 306 | 61% | 39% |
| Dover Buckland | 290 | 89% | 11% |
| Eriswell | 2 | 96% | 4% |
| Eriswell | 4 | 89% | 11% |
| Eriswell | 302 | 29% | 71% |
| Eriswell | 384 | 96% | 4% |
| Eriswell | 4238 | 7% | 92% |
| **Middle Anglo-Saxon** |  |  |  |
| Brandon | 785 | 36% | 64% |
| Brandon | 882 | 0% | 100% |
| Brandon | 883 | 100% | 0% |
| Brandon | 884 | 0% | 100% |
| Brandon | 889 | 0% | 100% |
| Brandon | 891 | 18% | 84% |
| Brandon | 892 | 21% | 79% |
| Brandon | 895 | 0% | 100% |
| Brandon | 2439 | 14% | 86% |
| Brandon | 2954 | 7% | 93% |
| Brandon | 3055 | 21% | 79% |
| Brandon | 3057 | 0% | 100% |
| Brandon | 3060 | 0% | 100% |
| Brandon | 3072 | 100% | 0% |
| Brandon | 3073 | 11% | 89% |
| Burwell | 56 | 96% | 4% |
| Burwell | 36 | 93% | 7% |
| Burwell | 16 | 89% | 11% |
| Burwell | 18 | 21% | 79% |
| Burwell | 19 | 100% | 0% |
| Burwell | 24 | 7% | 93% |
| Burwell | 25 | 7% | 93% |
| Burwell | 28 | 89% | 11% |
| Burwell | 29 | 36% | 64% |
| Burwell | 30 | 0% | 100% |
| Burwell | 32 | 32% | 68% |
| Burwell | 33 | 82% | 18% |
| Burwell | 37 | 96% | 4% |
| Burwell | 38 | 4% | 96% |
