# Supplementary material for: A 3D basicranial shape-based assessment of local and continental northwest European ancestry among 5th to 9th century CE Anglo-Saxons
Source: PLoS One. 2021 Jun 23;16(6):e0252477. doi: 10.1371/journal.pone.0252477 (PMC8221467; doi:10.1371/journal.pone.0252477)
Supplement: Attachment — Submitted filename: PONE-D-21-04061_reviewer (peer review).pdf [file pone.0252477.s003.pdf]

PLOS ONE

A 3D basicranial shape-based assessment of local and continental northwest European ancestry among 5th to 9th century CE Anglo-Saxons

--Manuscript Draft--

**Manuscript Number:**	PONE-D-21-04061
**Article Type:**	Research Article
**Full Title:**	A 3D basicranial shape-based assessment of local and continental northwest European ancestry among 5th to 9th century CE Anglo-Saxons
**Short Title:**	Basicranial shape and Anglo-Saxon ancestry
**Corresponding Author:**	Kimberly A Plomp, Ph.D.<br>Simon Fraser University<br>Burnaby, BC CANADA
**Keywords:**	Migration, colonization, cranial shape, geometric morphometrics, population history
**Abstract:**	The settlement of Great Britain by Germanic-speaking people from continental northwest Europe between the mid 5th and early 7th centuries CE has long been recognised as an important event, but uncertainty remains about the number of settlers and the nature of their relationship with the indigenous people of the island. The study reported here sought to shed light on these issues by using 3D shape analysis techniques to compare the cranial bases of Anglo-Saxon skeletons to those of skeletons from Great Britain that pre-date the Medieval period and skeletons from Denmark that date to the Iron Age and Viking Age. Analyses focused on Early Anglo-Saxon skeletons indicated that between two-thirds and three-quarters of Anglo-Saxon individuals were of continental northwest Europe ancestry, while between a quarter and one-third were of local ancestry. In contrast, analyses focused on Middle Anglo-Saxon skeletons suggested that 50-70% were of local ancestry, while 30-50% were of continental northwest Europe ancestry. The study suggests, therefore, that identity in Early Medieval Britain was similar to what it is today—inclusive and dynamic.
**Order of Authors:**	Kimberly A Plomp, Ph.D.
	Keith Dobney
	Mark Collard
**Additional Information:**	
**Question**	**Response**
**Financial Disclosure**<br><br>Enter a financial disclosure statement that describes the sources of funding for the work included in this submission. Review the submission guidelines for detailed requirements. View published research articles from *PLOS ONE* for specific examples.<br><br>This statement is required for submission and **will appear in the published article** if the submission is accepted. Please make sure it is accurate.	The study was supported by the European Union's Marie Skłodowska-Curie Actions program (KP - SAR10359), the Social Sciences and Humanities Research Council of Canada (MC - 895-2011-1009), the Canada Research Chairs Program (MC - 228117 and 231256), the Canada Foundation for Innovation (MC - 203808), the British Columbia Knowledge Development Fund (MC - 862-804231), and Simon Fraser University (MC - 14518).

Powered by Editorial Manager® and ProduXion Manager® from Aries Systems Corporation

**Unfunded studies**<br>Enter: *The author(s) received no specific funding for this work.*<br><br>**Funded studies**<br>Enter a statement with the following details:<br>• Initials of the authors who received each award<br>• Grant numbers awarded to each author<br>• The full name of each funder<br>• URL of each funder website<br>• Did the sponsors or funders play any role in the study design, data collection and analysis, decision to publish, or preparation of the manuscript?<br>• **NO** - Include this sentence at the end of your statement: *The funders had no role in study design, data collection and analysis, decision to publish, or preparation of the manuscript.*<br>• **YES** - Specify the role(s) played.<br><br>* typeset	
**Competing Interests**<br><br>Use the instructions below to enter a competing interest statement for this submission. On behalf of all authors, disclose any competing interests that could be perceived to bias this work—acknowledging all financial support and any other relevant financial or non-financial competing interests.<br><br>This statement is required for submission and **will appear in the published article** if the submission is accepted. Please make sure it is accurate and that any funding sources listed in your Funding Information later in the submission form are also declared in your Financial Disclosure statement.<br><br>View published research articles from *PLOS ONE* for specific examples.	The authors have declared that no competing interests exist.

Powered by Editorial Manager® and ProduXion Manager® from Aries Systems Corporation

**NO authors have competing interests**<br><br>Enter: *The authors have declared that no competing interests exist.*<br><br>**Authors with competing interests**<br><br>Enter competing interest details beginning with this statement:<br><br>*I have read the journal's policy and the authors of this manuscript have the following competing interests: [insert competing interests here]*<br><br>* typeset	
**Ethics Statement**<br><br>Enter an ethics statement for this submission. This statement is required if the study involved:<br><br>• Human participants<br>• Human specimens or tissue<br>• Vertebrate animals or cephalopods<br>• Vertebrate embryos or tissues<br>• Field research<br><br>Write "N/A" if the submission does not require an ethics statement.<br><br>General guidance is provided below. Consult the submission guidelines for detailed instructions. **Make sure that all information entered here is included in the Methods section of the manuscript.**	N/A

**Format for specific study types**<br><br>**Human Subject Research (involving human participants and/or tissue)**<br>• Give the name of the institutional review board or ethics committee that approved the study<br>• Include the approval number and/or a statement indicating approval of this research<br>• Indicate the form of consent obtained (written/oral) or the reason that consent was not obtained (e.g. the data were analyzed anonymously)<br><br>**Animal Research (involving vertebrate animals, embryos or tissues)**<br>• Provide the name of the Institutional Animal Care and Use Committee (IACUC) or other relevant ethics board that reviewed the study protocol, and indicate whether they approved this research or granted a formal waiver of ethical approval<br>• Include an approval number if one was obtained<br>• If the study involved *non-human primates*, add *additional details* about animal welfare and steps taken to ameliorate suffering<br>• If anesthesia, euthanasia, or any kind of animal sacrifice is part of the study, include briefly which substances and/or methods were applied<br><br>**Field Research**<br><br>Include the following details if this study involves the collection of plant, animal, or other materials from a natural setting:<br>• Field permit number<br>• Name of the institution or relevant body that granted permission	
**Data Availability**<br><br>Authors are required to make all data underlying the findings described fully available, without restriction, and from the time of publication. PLOS allows rare exceptions to address legal and ethical concerns. See the PLOS Data Policy and FAQ for detailed information.	Yes - all data are fully available without restriction

A Data Availability Statement describing where the data can be found is required at submission. Your answers to this question constitute the Data Availability Statement and **will be published in the article**, if accepted.<br><br>**Important:** Stating 'data available on request from the author' is not sufficient. If your data are only available upon request, select 'No' for the first question and explain your exceptional situation in the text box.<br><br>Do the authors confirm that all data underlying the findings described in their manuscript are fully available without restriction?	
**Describe where the data may be found in full sentences. If you are copying our sample text, replace any instances of XXX with the appropriate details.**<br><br>• If the data are **held or will be held in a public repository**, include URLs, accession numbers or DOIs. If this information will only be available after acceptance, indicate this by ticking the box below. For example: *All XXX files are available from the XXX database (accession number(s) XXX, XXX.).*<br>• If the data are all contained **within the manuscript and/or Supporting Information files**, enter the following: *All relevant data are within the manuscript and its Supporting Information files.*<br>• If neither of these applies but you are able to provide **details of access elsewhere**, with or without limitations, please do so. For example:<br><br>*Data cannot be shared publicly because of [XXX]. Data are available from the XXX Institutional Data Access / Ethics Committee (contact via XXX) for researchers who meet the criteria for access to confidential data.*<br><br>*The data underlying the results presented in the study are available from (include the name of the third party*	All relevant data are within the manuscript and its Supporting Information files.

and contact information or URL).<br>• This text is appropriate if the data are owned by a third party and authors do not have permission to share the data.<br><br>* typeset	
Additional data availability information:	

Powered by Editorial Manager® and ProduXion Manager® from Aries Systems Corporation

Manuscript Click here to access/download;Manuscript;Manuscript.docx

A 3D basicranial shape-based assessment of local and continental northwest European ancestry among 5th to 9th century CE Anglo-Saxons

Plomp K.A.[1,2,*], Dobney K.[1,2,3], Collard M.[1,*]

[1]Department of Archaeology, Simon Fraser University, 8888 University Drive, Burnaby, British Columbia, V5A 1S6, Canada.

[2]Department of Archaeology, Classics, and Egyptology, University of Liverpool, 12-14 Abercromby Square, Liverpool, L68 7WZ, UK.

[3]School of Historical and Philosophical Inquiry, University of Sydney, A18 Brennan MacCallum, Sydney, NSW 2006, Australia.

Abstract

The settlement of Great Britain by Germanic-speaking people from continental northwest Europe between the mid 5th and early 7th centuries CE has long been recognised as an important event, but uncertainty remains about the number of settlers and the nature of their relationship with the indigenous people of the island. The study reported here sought to shed light on these issues by using 3D shape analysis techniques to compare the cranial bases of Anglo-Saxon skeletons to those of skeletons from Great Britain that pre-date the Medieval period and skeletons from Denmark that date to the Iron Age and Viking Age. Analyses focused on Early Anglo-Saxon skeletons indicated that between two-thirds and three-quarters of Anglo-Saxon individuals were of continental northwest Europe ancestry, while between a quarter and one-third were of local ancestry. In contrast, analyses focused on Middle Anglo-Saxon skeletons suggested that 50-70% were of local ancestry, while 30-50% were of continental northwest Europe ancestry. The study suggests, therefore, that identity in Early Medieval Britain was similar to what it is today—inclusive and dynamic.

Keywords: Migration, colonization, cranial shape, geometric morphometrics, population history

Introduction

The settlement of large parts of the island of Great Britain by Germanic-speaking people from continental northwest Europe between the mid 5th and early 7th centuries CE has long been recognised as an important event, leading as it did to the formation of the ethnic group commonly referred to as the Anglo-Saxons[1], the development of the English language, and the formation of the Kingdom of England and, eventually, the United Kingdom [1-4]. Not surprisingly, therefore, this episode has been the subject of a considerable amount of research (see Hardland [5] for a recent review). Despite this, uncertainty remains about the number of settlers and the nature of their relationship with the indigenous people of the island, especially the Romano-British.

Traditionally, knowledge of the settlement of Britain by the Angles, Saxons, Jutes, and Frisians relied on two historical texts, *Historia Ecclesiastica Gentis Anglorum* and the *Anglo-Saxon Chronicle*. Written by the Venerable Bede, *Historia Ecclesiastica* is thought to have been completed in 731 CE. The original version of the *Anglo-Saxon Chronicle* was created late in the 9th century CE, and copies of it were independently updated until at least 1154 CE. Both of these documents describe a mass invasion and a rapid replacement of the indigenous population [6,7,8].

This picture has been challenged by archaeologists. They have argued that the archaeological record reveals that the changes associated with the arrival of the Germanic-speaking settlers in

[1]The appropriate name for the ethnic group in question has long been a matter of contention. While they are usually referred to as the 'Anglo-Saxons' in the present day, it has been pointed out that this name was not often used by them [15]. They seem to have preferred the term 'English' (*Angli*, *Anglici*). We have elected to use the term 'Anglo-Saxons' for ease of communication.

Britain occurred relatively slowly, and that this is inconsistent with the idea that the settlers replaced the Romano-British [9-11]. In line with this, analyses of oxygen and strontium isotopes from Anglo-Saxon skeletons have found that only a small minority of the sampled individuals were from the Continent [12-14].

Geneticists have also sought to shed light on these issues, but the results they have obtained are highly variable. A comparison of the ancestry estimates reported by Weale et al. [2], Leslie et al. [16], and Schiffels et al. [17] illustrates this. Based on analyses of modern Y chromosome DNA, Weale et al. [2] concluded that 50-100% of men in central England have male ancestors from continental northwest Europe. Leslie et al.'s [16] analyses of modern genomes suggested that 10-40% of people from central and southern England have continental northwest European ancestry. Schiffels et al. [17] analysed the genomes of ten Iron Age and Anglo-Saxon individuals from England. They found that the Anglo-Saxon individuals were closely related to modern Danish and Dutch people and estimated that introgression from continental northwest Europe accounted for 38% of the ancestry of people currently living in eastern England.

Here, we report a study designed to bring a new line of evidence to bear on the issue. We used three-dimensional (3D) shape analysis techniques to compare the cranial bases of Anglo-Saxon skeletons to those of skeletons from Great Britain that pre-date the Medieval period and skeletons from Denmark that date to the Iron Age and Viking Age. We focused on the basicranium because previous studies have shown that the 3D shape of this region of the skull can be informative about relatedness among human populations (e.g. [18-21]). The shape analysis techniques we utilised are collectively referred to as 'Geometric Morphometrics' [22-24]. These techniques

have been used extensively by palaeoanthropologists to tackle comparable problems (e.g. [25,26]). The goal of the analyses was to estimate the percentage of Anglo-Saxon individuals who were of British ancestry and the percentage who were of continental northwest European ancestry.

Materials and methods

The sample is summarized in Table 1 and the locations of the sites are shown in Figure 1. Further information about the specimens can be found in the Supplementary Information.

We recorded data on a total of 237 individuals. Only adult crania were measured to avoid the confounding effects of ontogeny; specimens were judged to be adult on the basis of dental eruption and epiphyseal fusion. Both males and females were included in the sample, with sex being determined on the basis of pelvic and cranial morphology [27].

Eighty-nine of the specimens come from five Anglo-Saxon cemeteries: Brandon in Suffolk, Breedon-on-the-Hill in Leicestershire, Burwell in Cambridgeshire, Dover Buckland in Kent, and Eriswell in Suffolk. Breedon-on-the-Hill, Dover Buckland, and Eirswell date to the Early Anglo-Saxon Period (410-660 CE), while Brandon and Burwell date to Middle Anglo-Saxon Period (660-889 CE). Hereinafter, we will refer to the individuals from Breedon-on-the-Hill, Dover Buckland, and Eirswell as Early Anglo-Saxons, and the individuals from Brandon and Burwell as Middle Anglo-Saxons.

Another 101 of the specimens are from pre-Medieval sites in England. The sites in question are Poundbury and Maiden Castle in Dorset, and Hallet’s Garage in Kent. Poundbury and Hallet’s Garage are both of Romano-British date (43-410 CE), while Maiden Castle dates to the preceding Iron Age (800 BCE-43 CE).

The remaining 46 individuals are from Iron Age and Viking Age various sites in Denmark. We focused on specimens from Denmark partly because Schiffels et al.’s [17] analyses indicated that the early English specimens in their sample from Cambridgeshire were genetically closer to modern Danish people, and partly because they are better preserved than similarly dated specimens from the other main potential source areas, northern Germany and the Netherlands.

We used photogrammetry to generate 3D models of the crania. Each cranium was photographed 150 times with an eight-megapixel digital single-lens reflex Canon EOS 77D camera mounted with a Canon 50mm lens. The cranium was placed on a PalaeoPi rotating table and then photographs were shot at intervals of approximately 10°, as per Evin et al. [28] Subsequently, the photographs for a single cranium were converted into a 3D model in Metashape. The photographs were aligned at the ‘high’ accuracy level and 3D depth maps generated. After this, the 3D depth maps were used to create a mesh model of each cranium.

To digitize the 3D shape of the specimens’ cranial bases, we imported the mesh models into MorphoDig [29] and recorded the 3D Cartesian coordinates of a total of 34 anatomical landmarks (Figure 2). The landmarks were chosen based on those used by Harvati and Weaver

[18] to capture the shape of the basicranium. According to Bookstein's [30] widely used scheme, seven of the landmarks are Type I landmarks and 27 are Type II landmarks.

Intra-observer error was assessed in the manner outlined by Neubauer et al. [31,32]. We digitised a single cranium ten times and then used Morphologika [33] to compare the greatest Procrustes distance between the ten repeated landmark configurations with the smallest Procrustes distance between the non-repeated landmark configurations representing all the crania. The smallest distance between the non-repeated cranium was almost double the greatest distance between the repeated crania. According to Neubauer et al. [31.32], this amount of error is unlikely to influence the analysis of the shape variance of the sample.

After assessing the intra-observer error, we divided the dataset by sex. We did so to avoid sexual dimorphism influencing the results.

We then sought to minimise the impact of a number of other potential confounding factors on each sex-specific dataset. Following Klingenberg et al. [33], we reflected and re-labelled the landmark coordinates and subjected the data to generalised Procrustes analysis, which removes translational and rotational effects and scales the configurations to centroid size. We removed asymmetry by calculating the average Procrustes coordinates between the original and reflected landmarks. These procedures were carried out in MorphoJ [33].

Having minimised the confounding effects of translation, rotation, size, and asymmetry, we divided the specimens in the female dataset into four groups: Early Anglo-Saxons, Middle Anglo-Saxons, Pre-Medieval British, and Danish. We then did the same for the male dataset.

Subsequently, we carried out three sets of analyses. In the first, we explored the shape variance among the four groups. We did this by applying canonical variates analysis (CVA) to the Procrustes coordinates. We conducted two analyses, one that concentrated on the female dataset and one that focused on the male dataset. The CVAs were performed in MorphoJ [33].

In the second set of analyses, we investigated whether there were statistically significant shape differences among the four groups of specimens. The primary goal of these analyses was to ensure that the two potential source groups, the Pre-Medieval British and Danish, differ, because this was a prerequisite for making meaningful statements about the ancestry of the Anglo-Saxon individuals. We first subjected each dataset to principal components analysis (PCA) followed by the principal component (PC) reduction procedure developed by Baylac and Frieβ [34]. This procedure aims to reduce noise from PCs that account for little variance while still retaining all relevant shape information. It tackles this optimization problem by progressively adding PCs into the analysis until the cross-validation percentage begins to drop. Thereafter, we applied MANOVA to the retained PC scores. As before, the female and male specimens were analysed separately. The PCAs were performed in MorphoJ [33]; the CVP analyses and MANOVAs were performed in R [35].

In the third set of analyses, we assessed the relative contribution of the two potential source groups to the ancestry of the two Anglo-Saxon groups. We did this by applying linear discriminant analysis (LDA) to the PCs used in the previous set of analyses. Following Evin et al. [36], we designated the potential source groups as the known samples and then directed the LDA to indicate which of the potential source groups the Anglo-Saxon individuals most likely belonged. We repeated this process 100 times for each Anglo-Saxon individual and then calculated the average attribution percentages. An individual was deemed to be attributed to a given source group if the average percentage for that group was ≥55%. If all the average attribution percentages for an individual were ≤54%, the specimen was deemed to be unattributable. Thereafter, we calculated the percentage of Early Anglo-Saxons and Middle Anglo-Saxons that were attributed to each of the potential source groups. Once again, the female and male specimens were analysed separately. The LDAs were performed in R.

Results

The CVA of the female specimens yielded three CVs. CV1 accounted for 58% of the variance, CV2 for 27%, and CV3 for 14%.

Figure 3a shows the values for CV1 and CV2 for the female specimens plotted against each other. The Pre-Medieval British and Danish overlap nearly completely on CV2 but only partially on CV1. On CV1, the bulk of the Pre-Medieval British specimens are located more positively than the majority of the Danish ones. The Early Anglo-Saxons overlap substantially with both the Pre-Medieval British and the Danish on CV2, but only with the Danish on CV1. The Middle Anglo-Saxons overlap substantially with the Early Anglo-Saxons on CV1 but do not overlap

with the Pre-Medieval British or Danish on the same CV. In contrast, the Middle Anglo-Saxons overlap somewhat with the Pre-Medieval British and Danish individuals on CV2; the overlap with the Pre-Medieval British individuals is greater than the overlap with the Danish ~~ones~~.

The values for CV2 and CV3 for the females are plotted against each other in Figure 3b. The Pre-Medieval British and Danish individuals overlap almost entirely on CV2 but only a small amount on CV3. On CV3, the middle of the distribution of the Pre-Medieval British is closer to the negative end of the CV than the middle of the distribution of the Danish. The Early Anglo-Saxons overlap substantially with the Pre-Medieval British on both CVs. In contrast, while the Early Anglo-Saxons overlap with the Danish individuals on CV2, they barely overlap with them on CV3. The Middle Anglo-Saxons have a different relationship with the Pre-Medieval British and Danish individuals. They barely overlap with the Danish on either CV. They overlap markedly more with the Pre-Medieval British on CV2 and even more with them on CV3. The Middle Anglo-Saxons overlap with Early Anglo-Saxons on CV3 but are separated from them on CV2.

The CVA of the male specimens also produced three CVs. CV1 accounted for 44% of the variation, CV2 for 37%, and CV3 for 19%.

Figure 4a shows the values for CV1 and CV2 for the male specimens plotted against each other. The Pre-Medieval British and Danish individuals overlap on both CVs, although to a lesser extent on CV1. On both CVs, the centre of the distribution of Pre-Medieval British specimens is closer to the positive end of the CV than the centre of the distribution of the Danish individuals.

The Early Anglo-Saxons overlap substantially with both the Pre-Medieval British and Danish on CV2. The situation is different when we look at CV1. On this CV, the Early Anglo-Saxons overlap with the Danish individuals to a considerable extent, but barely overlap with the Pre-Medieval British ones. The Middle Anglo-Saxons overlap substantially with the Pre-Medieval British, Danish, and Early Anglo-Saxon individuals on CV1. On CV2, they do not overlap at all with the Danish individuals and only slightly with the Pre-Medieval British and Early Anglo-Saxon ones.

The CV2 and CV3 values for the males are plotted against each other in Figure 4b. On CV2, the Danish specimens are positioned towards the negative end of the CV and the Pre-Medieval British ones towards the centre of the CV. The Early Anglo-Saxons overlap substantially with both the Danish and Pre-Medieval British ~~ones~~ on CV2. On CV3, Early Anglo-Saxons overlap to a great extent with the Pre-Medieval British individuals but only partially with the Danish ~~ones~~. The Middle Anglo-Saxons are distributed in such a way that do not overlap with the Danish individuals on CV2. Instead, they partially overlap with the Pre-Medieval British individuals and extend to the positive end of the CV. On CV3, the Middle Anglo-Saxons overlap substantially with both the Pre-Medieval British and Danish individuals, although the overlap is greater in the case of the Pre-Medieval British ones. The Early Anglo-Saxons and Middle Anglo-Saxons overlap extensively on CV3, but barely overlap on CV2.

The PC reduction procedure retained 10 PCs for the female individuals. Seventy percent of the total shape variance was accounted for by these PCs. The female MANOVA indicated that the

shapes of the basicrania of the Pre-Medieval British and Danish specimens are significantly different from each other (λ 0.4112, F=2.002, p=0.038). In addition, the Early Anglo-Saxon females were statistically different from both the Middle Anglo-Saxon females (λ 0.560, F=2.282, p=0.041) and the Pre-Medieval British females (λ 0.675, F=3.560, p<0.001). However, they were not significantly different from the Danish females (λ 0.755, F=0.975, p=0.485). The Middle Anglo-Saxon females were statistically different from the Pre-Medieval British females (λ 0.565, F=2.151, p=0.006) but not the Danish females (λ 0.295, F=1.768, p=0.060).

Twenty-one PCs were retained for the male specimens by the PC reduction procedure. These PCs accounted for 88% of the total shape variance. As was the case with the female MANOVA, the male MANOVA indicated that there is a significant difference in basicranial shape between the Pre-Medieval British and Danish groups (λ 0.3656, F=2.7669, p=0.002). The Early Anglo-Saxon males were statistically different from the Middle Anglo-Saxon (λ 0.356, F=3.362, p<0.001), Pre-Medieval British (λ 0.492, F=3.891, p<0.001), and Danish (λ 0.349, F=2.668, p=0.007) males. In addition, the Middle Anglo-Saxon males were statistically different from the Pre-Medieval British (λ 0.595, F=2.855, p<0.001) and Danish (λ 0.356, F=3.362, p<0.001) males.

Table 2 shows the percentage of the Early Anglo-Saxons and Middle Anglo-Saxons for whom the predicted source population was the Pre-Medieval British group, the percentage for whom the predicted source population was the Danish group, and the percentage deemed unattributable. The individual probabilities are listed in Table S1.

In the female LDA analysis, 63% of the Early Anglo-Saxons were attributed to the Danish group, and 26% to the Pre-Medieval British group. The remaining 11% were deemed to be of uncertain ancestry. Thus, nearly two-thirds of the Early Anglo-Saxons were suggested to be of non-local ancestry, while a quarter were deemed to be of local ancestry. The pattern was different for the Middle Anglo-Saxons. Fifty-four percent of these individuals were attributed to the Pre-Medieval British group; 39% were attributed to the Danish group; and 8% were deemed to be of uncertain ancestry. In other words, just over half of the Middle Anglo-Saxons were suggested to be of local ancestry and just under half were suggested to be of non-local ancestry.

In the male LDA analysis, 65% of the Early Anglo-Saxons were attributed to the Danish group, and 25% to the Pre-Medieval British group. The remaining 10% were deemed to be of uncertain ancestry. As was the case with the female LDA analysis, the pattern was different for the Middle Anglo-Saxons. Sixty-nine percent of these were attributed to the Pre-Medieval British group, and 31% to the Danish group. As such, the results of the male LDA analysis were similar to those of the female LDA analysis. They also indicated that nearly two-thirds of the Early Anglo-Saxons were of non-local ancestry while a quarter were of local ancestry. Also like the female data, the male data indicate that the pattern reversed with the Middle Anglo-Saxons, with the majority of them being suggested to have been of local ancestry and the minority of non-local ancestry.

Table 2. Summary of the results of the Linear Discriminant Analyses. The table shows the percentage of Anglo-Saxon individuals attributed to the Danish and Pre-Medieval British groups, and the percentage deemed to be of uncertain ancestry. See the Materials and Methods for details of the criterion that was used to identify the latter cases.

	**Females**			**Males**		
	Danish	*Pre-Medieval British*	*Unknown*	*Danish*	*Pre-Medieval British*	*Unknown*
**Early Anglo-Saxons**	63%	26%	11%	65%	30%	5%
**Middle Anglo-Saxons**	39%	53%	8%	31%	69%	-

Discussion

In the study reported here, we used 3D shape data from the basicranial portion of the skull to infer the ancestry of a large sample of Anglo-Saxon skeletons from southern Britain and therefore shed light on the scale of the migration from continental northwest Europe that occurred between the mid 5th and early 7th centuries CE. Analyses that focused on Early Anglo-Saxon skeletons indicated that between two-thirds and three-quarters of them were likely of continental northwest Europe ancestry, while between a quarter and one-third were of local ancestry. Analyses that focused on Middle Anglo-Saxon skeletons returned substantially different results. They indicated that 50-70% of the Anglo-Saxon individuals were likely of local ancestry, while 30-50% of them were likely of continental northwest Europe ancestry.

To ensure our decision to keep the female and male specimens separate did not skew our results, we ran a supplementary analysis. We created a mixed-sex dataset and then subjected it to LDA in the manner described earlier. Of the 47 Early Anglo-Saxons, 72% were assigned to the Danish group, 23% to the Pre-Medieval British group, and 4% were deemed unattributable. Of the 42 Middle Anglo-Saxons, 52% were assigned to the Pre-Medieval British group, 45% to the Danish group, and 2% were deemed unattributable. These results are similar to those obtained in the sex-specific LDAs, which indicates that our decision to keep the female and male specimens separate did not skew our results.

~~As explained previously,~~ three other lines of evidence have been used to investigate the relative number of individuals of local ancestry and individuals of continental northwest European ancestry among the Anglo-Saxons: historical texts, isotopes, and DNA. ~~To reiterate,~~ the relevant

historical texts, the *Historia Ecclesiastica* and the *Anglo-Saxon Chronicles*, describe a wholesale replacement of the native population by a large number of Germanic-speaking people from continental northwest Europe. A plausible explanation for the discrepancy between our results and the historical texts is that the latter are simply inaccurate. As Hamerow [37] and Thomas et al. [38] have pointed out, the *Historia Ecclesiastica* and the *Anglo-Saxon Chronicles* were both written several centuries after the events in question and therefore it is feasible that their authors greatly exaggerated the number of settlers. One possibility is that continental northwest European ancestry was more prestigious than local ancestry and that prompted the majority of later Anglo-Saxons to claim to have continental northwest European ancestors. Another interesting possibility is raised by Reynolds' [15] work. She argued that Medieval people believed that humans were "divided into 'peoples' (*gentes*, *nationes*, *populi*) of common biological descent and culture who normally and naturally formed separate political units". It is not hard to see how this belief could have led to the creation of a founding myth in which the Anglo-Saxons were solely the descendants of Germanic-speaking settlers.

A number of isotopic studies have focused on Anglo-Saxon skeletons. For example, Budd et al. [12] analysed oxygen isotope ratios in 32 individuals from the cemetery of West Heslerton, North Yorkshire, and concluded approximately half of the samples analysed were from other areas of Britain, while four females appeared to originate from Scandinavia or Baltic Europe. Subsequently, Hughes et al. [13] extracted strontium and oxygen isotopes from the remains of 19 Anglo-Saxon individuals from Berinsfield, Oxfordshire. Four of 19 individuals were found to have isotope values indicating they did not spend their childhood in the area. One of these individuals was deemed to be from mainland Europe, while the provenance of the other three was

deemed uncertain; the most the authors could say was that they were ‘not local’. In line with their previous results, Hughes et al. [13] identified seven individuals of 19 from the Early Anglo-Saxon cemetery in Eastbourne, Sussex, with non-local strontium isotope values. Four of these seven were interpreted to be from mainland Europe. Thus, the isotope studies suggest that only a small number of Anglo-Saxons were from mainland Europe.

There would seem to be several potential explanations for the discrepancy between the results of the isotope studies and our study’s finding. To begin with, it is possible that the discrepancy is more apparent than real. Analyses of oxygen and strontium isotopes extracted from teeth shed light on where an individual grew up rather than on their ancestry. So, it is feasible that some, if not all, of the individuals who were deemed to be local had parents raised on the Continent but were themselves raised in southern Britain, i.e. they were second-generation settlers. If this were the case, then the results reported here and those of the isotope studies are not inconsistent. A second possibility is that there was regional variation in the number of Germanic-speaking settlers. None of the Anglo-Saxon skeletons we analysed are among those sampled in the isotope studies. So, it is possible that both sets of results are correct and that the number of Germanic-speaking settlers was simply higher in the areas of southern Britain represented in the isotope studies than the area represented in the present study. A third possibility is that the isotope studies underestimated the number of individuals from the Continent among the Anglo-Saxons. All three studies identified additional non-local individuals in their samples. Some of these individuals were hypothesised to be from elsewhere in the British Isles, while others were deemed to be of uncertain provenance. However, there is a problem with these assessments. They depend on good data on landscape $^{87}Sr/^{86}Sr$ isotope ratio variability in the regions of interest. As Bataille et al. [39]

demonstrate, while the British Isles and the homeland of incoming people have been well sampled for $^{87}Sr/^{86}Sr$ isotope ratios, the data for the other areas of interest in continental Europe are limited and patchy. This raises the possibility that some of individuals who were identified as having been from elsewhere in the British Isles could in fact have been from continental northwest Europe. The same holds for the individuals that the authors were unable to provenance.

The earliest of the DNA studies to have focused on the origins of the Anglo-Saxons estimated that 50-100% of males in England have mainland European ancestry [2]. Subsequent studies have estimated the percentage of mainland European ancestry in England to be between 10% and 73% [3,16,17]. Thus, in contrast to the situation with the historical texts and perhaps with the isotope data, our results are not inconsistent with the available DNA evidence regarding the ancestry of the Anglo-Saxons. Our estimate of between two-thirds and three-quarters of the Early Anglo-Saxons having been of continental northwest Europe ancestry falls comfortably within the range of estimates obtained in the DNA studies. So does our estimate of between a third and a half of Middle Anglo-Saxons having been of continental northwest Europe ancestry.

It is intriguing that our results indicate that the number of Anglo-Saxons of continental northwest European ancestry decreased from about two-thirds in the Early Anglo-Saxon Period to around one-third in the Middle Anglo-Saxon Period. To the best of our knowledge, a marked change in relative numbers of locals and non-locals between these periods has not been identified before. The historical texts do not mention one; nor do the isotope and DNA studies. There are a number of potential explanations for such a change. To begin with, it could mean that there was an increase in the number of local people adopting the Anglo-Saxon identity from the third to the

seventh century. Alternatively, it could mean that mass migration of peoples from mainland Europe had ceased and there was an increase in intermarriage between people of continental northwest European ancestry and those of local ancestry. A third possibility is that there was still migration from mainland Europe after 660 CE but at a much lower level than in the preceding period. Lastly, it is possible that the increase in the percentage of individuals of local ancestry was a consequence of those with local ancestry out-reproducing those of continental northwest European ancestry. Determining which, if any, of these hypotheses is correct will require further research.

Two other possibilities for future research suggest themselves. One is to repeat the study with a more comprehensive sample. In particular, it would also be useful to include individuals from regions of Britain not represented in the present study (see Figure 1). This would allow the hypothesis that there was regional variation in the relative number of Anglo-Saxons of continental northwest European and local ancestry to be evaluated. The other obvious next step is to repeat the study with data from one or more other regions of the skull. We focused on the basicranium because previous analyses have found that 3D shape correlates significantly with among-population genetic distance in humans, but studies in palaeoanthropology have suggested that there are no major differences among the cranial regions with regard to inferring ancestry [40]. With this in mind, it would be useful to apply 3D geometric morphometric techniques to the faces, cranial vaults, and/or mandibles of a large sample of suitably dated individuals. Doing so could allow one of the major shortcomings of the present study to be overcome—namely, the inability to include specimens from Germany and the Netherlands due to poor preservation of the cranial base.

Conclusions

The study reported here focused on an important unresolved issue in British history—the relative number of individuals of local ancestry and individuals of continental northwest European ancestry among the Anglo-Saxons. Existing lines of evidence do not agree on this issue. Historical texts indicate that the vast majority of Anglo-Saxons were of continental northwest European ancestry, while stable isotope analyses suggest that there were in fact only a few individuals from mainland Europe among the Anglo-Saxons. Unfortunately, analyses of DNA have not clarified the situation. The results that have been reported to date are highly variable, with estimates of the percentage of continental northwest European ancestry in England ranging between 10% and 100%, depending on the study.

We sought to reduce the uncertainty by comparing the basicrania of individuals from five Anglo-Saxon cemeteries to the basicrania of individuals from sites in the British Isles that predate the Medieval period, and sites in Denmark that date to the Iron Age and Viking Age. Denmark was the homeland of two of the Germanic-speaking groups whose members settled in southern Britain between the mid 5th and early 7th centuries CE and contributed to the formation of the Anglo-Saxon ethnic group. Thus, the comparison allowed us to estimate the percentage of Anglo-Saxon individuals who were of local ancestry and the percentage who were of continental northwest European ancestry.

The analyses we carried out that focused on skeletons from the Early Anglo-Saxon Period (410-660 CE) indicated that between two-thirds and three-quarters of the Anglo-Saxon individuals

were likely of continental northwest Europe ancestry, while between a quarter and one-third of them were people of local ancestry. The analyses we conducted that focused on individuals from the Middle Anglo-Saxon Period (660-889 CE) returned substantially different results. They indicated that 50-70% of the Anglo-Saxon individuals were likely of local ancestry, while 30-50% of them were likely of continental northwest Europe ancestry.

Our study suggests, therefore, that the Anglo-Saxon ethnic group comprised many individuals of continental northwest European ancestry but also many of local ancestry. It also suggests that relative number of individuals of continental northwest European ancestry and individuals of local ancestry changed through time. In the Early Anglo-Saxon Period, individuals of continental northwest European ancestry heavily outnumbered individuals of local ancestry, but by the Middle Anglo-Saxon Period, those of local ancestry outnumbered those of continental northwest European ancestry. Intriguingly, it appears that identity in Early Medieval Britain was similar to what it is today—inclusive and dynamic.

Declarations

Ethics approval: The study analysed archaeological human skeletons and did not require Ethics Approval.

Consent for publication: Not applicable.

Availability of data and materials: The landmark data are made available in the supplementary documents.

Funding

The study was supported by the European Union's Marie Skłodowska-Curie Actions program (SAR10359), the Social Sciences and Humanities Research Council of Canada (895-2011-1009), the Canada Research Chairs Program (228117 and 231256), the Canada Foundation for Innovation (203808), the British Columbia Knowledge Development Fund (862-804231), and Simon Fraser University (14518).

Authors' contributions

KAP, KD, and MC designed the study. KAP, MC, and KD obtained funding for the study. KAP collected and analysed the data. KAP and MC interpreted the results and drafted the manuscript. KAP, KD, and MC edited the manuscript. All authors read and approved the final manuscript.

Acknowledgements

We offer special thanks to Carly Ameen and Allowen Evin for their assistance with the analyses. We also thank Neil Price for his advice regarding the manuscript. The following institutions

kindly provided access to the collections used in the study: University of Oslo, Oslo, Norway; University of Copenhagen, Copenhagen, Denmark; the National Museum of Natural History, London, UK; the Duckworth Laboratory at the University of Cambridge, Cambridge, UK.

References

1) Crabtree PJ. Sheep, horses, swine, and kine: a zooarchaeological perspective of Anglo-Saxon settlement of England. J Field Archaeol. 1989;16: 205-213.

2) Weale ME, Weiss DA, Jager RF, Bradman N, Thomas MG. Y chromosome evidence for Anglo-Saxon mass migration. Mol Biol Evol. 2002;19: 1008-1021.

3) Capelli C, Redhead N, Abernethy JK, Gratix F, Wilson JF, Moen T, Hervig T, Richards M, Stumpf MPH, Underhill PA, Bradshaw P, Shaha A, Thomas MG, Bradman N, Goldstein DB. AY chromosome census of the British Isles. Cur Biol. 2003;13: 979-984.

4) Härke H. Anglo-Saxon immigration and ethnogenesis. Med Archaeol. 2011;55: 1-28.

5) Hardland JM. Rethinking ethnicity and otherness in Early Anglo-Saxon England. Med Worlds. 2017;5: 113-142.

6) Stenton FM. The Oxford history of England. Oxford; Clarendon Press. 1947.

7) Whitelock D. English historical documents, c 500-1042. London; Eyre Methuen. 1979.

8) Arnold CJ. Roman Britain to Saxon England. London; Croom Helm. 1984.

9) Lucy S. From Pots to People: Two Hundred Years of Anglo-Saxon Archaeology. In: Hough C, Lowe KA, editors. Lastworda Betst: essays in memory of Christine E. Fell with her unpublished writings. Donnington: Paul Watkins; 2002: 144-148.

10) Lucy S. The early Anglo-Saxon cemeteries of East Yorkshire: an analysis and interpretation. Oxford: BAR 272; 1998.

11) Hills C. *Origins of the English*. London: Bloomsbury Publishing; 2003.

12) Budd P, Chenery C, Montgomery J, Evans J, Powlesland D. Anglo-Saxon residential mobility at West Heslerton, North Yorkshire, UK, from combined O- and SR-isotope analysis. In: Holland G, Tanner SD, editors. Plasma source mass spectrometry: applications and emerging technologies. London: Royal Society of Chemistry; 2003: 195-208.

13) Hughes SS, Millard AR, Lucy SJ, Chenery CA, Evans JA, Nowell G, Pearson DG. Anglo-Saxon origins investigated by isotopic analysis of burials from Berinsfield, Oxfordshire, UK. J Archaeol Sci. 2014;42: 81-92.

14) Hughes SS, Millard AR, Chenery CA, Nowell G, Pearson DG. Isotopic analysis of burials from the early Anglo-Saxon cemetery at Eastbourne, Sussex, UK. J Archaeol Sci Rep. 2018;19: 513-525.

15) Reynolds S. What do we mean by "Anglo-Saxon" and "Anglo-Saxons"? J Brit St. 1985;24: 395-414.

16) Leslie S, Winney B, Hellenthal G, Davison D, Boumertit A, Day T, Hutnik K, Royrvik EC, Cunliffe B, Wellcome Trust Case Control Consortium 2, International Multiple Sclerosis Genetics Consortium, Lawson DJ, Falush D, Freeman C, Pirinen M, Myers S, Robinson M, Donnelly P, Bodmer W. The fine-scale genetic structure of the British population. Nature. 2015;519: 309-314.

17) Schiffels S, Haak W, Paajanen P, Llamas B, Popescu E, Loe L, Clarke R, Lyons A, Mortimer R, Sayer D, Tyler-Smith A, Coooper A, Durbin R. Iron Age and Anglo-Saxon genomes from East England reveal British migration history. Nature Communications. 2016;7: 10408.

18) Harvati K, Weaver TD. Human cranial anatomy and the differential preservation of population history and climate signatures. Anat Rec. 2006;288A: 1225-1233.

19) Smith HF. Which cranial regions reflect molecular distances reliably in humans? Evidence from three-dimensional morphology. Am J Hum Biol. 2009;21: 36-47.

20) von Cramon-Taudadel, N. Congruence of individual cranial bone morphology and neutral molecular affinity patterns in modern humans. Am J Phys Anthropol. 2009;140: 205-215.

21) Smith HF. The role of genetic drift in shaping modern human cranial evolution: a test using microevolutionary modeling. Int J Evol Biol. 2011: 145262.

22) Mitteroecker P, Gunz P. Advances in geometric morphometrics. Evol Biol. 2009;36: 235-247.

23) O'Higgins P, Jones N. Facial growth in Cercobus torquatus: an application of three-dimensional geometric morphometric techniques to the study of morphological variation. J Anat. 1998;193: 251-272.

24) Slice D. Geometric morphometrics. Ann Rev Anthropol. 2007;36: 261-281.

25) von Cramon-Taubadel N, Smith HF. The relative congruence of cranial and genetic estimates of hominoid taxon relationships: implications for the reconstruction of hominin phylogeny. J Hum Evol, 2012;62: 640-653.

26) Zichello JM, Baab KL, McNulty KP, Raxworthy CJ, Steiper ME. Hominoid intraspecific cranial variation mirrors neutral genetic diversity. Proc Nat Ac Sci. 2018;6: 11501-11506**.**

27) Buikstra J, Ubelaker D. Standards for data collection from human skeletal remains. Fayetteville: Arkansas Archeological Survey. 1994.

28) Evin A, Souter T, Hulme-Beaman A, Ameen C, Allen R, Viacava P, Larson G, Cucchi T, Dobney K. The use of close-range photogrammetry in zooarchaeology: creating accurate 3D models of wolf crania to study dog domestication. J Archaeol Sci Rep. 2016;9: 87-93.

29) Lebrun R. MorphoDig, An Open-Source 3D Freeware Dedicated to Biology. Montpellier; Institut des Sciences de l'Evolution Université de Montpellier. 2018. URL: https://morphomuseum.com/morphodig.

30) Bookstein F. Landmark methods for forms without landmarks: morphometrics of group differences in outline shape. Med Image Anal. 1997;1: 225-243.

31) Neubauer S, Gunz P, Hublin JJ. The patterns of endocranial ontogenetic shape changes in humans. J Anat. 2009;215: 240-255.

32) Neubauer S, Gunz P, Hublin JJ. Endocranial shape changes during growth in chimpanzees and humans: a morphometric analysis of unique and shared aspects. J Hum Evol. 2010;59: 555-566.

33) Klingenberg CP, Barluenga M, Meyer A. Shape analysis of symmetric structures: Quantifying variation among individuals and asymmetry. Evolution. 2002;56: 1909-1920.

34) Baylac M, Frieb M. Fourier descriptors, Procrustes superimposition, and data dimensionality: An example of cranial shape analysis in modern human populations. In: Slice D, editor. Modern Morphometrics in Physical Anthropology, Part 1 Theory and Methods. New York; Springer. 2005.

35) R Development Core Team. R: A Language and Environment for Statistical Computing. R Foundation for Statistical Computing. Vienna. 2018. URL: https://www.r-project.org/.

36) Evin A, Girdland Flink L, Balasescu A, Popovici D, Andreescu, Bailey D, Mirea P, Lazar C, Boroneant A, Bonsall C, Strand Vidarsdottir U, Brehard S, Tresset A, Cucchi T, Larson G, Dobney K. Roy Soc Phil Trans B. 2015; 370: 20130616.

37) Hamerow H. Migration theory and the Anglo-Saxon “identity crisis”. In: Chapman J, Hamerow H, editors. Migrations and invasions in archaeological explanation. Oxford; BAR International Series. 1997.

38) Thomas MG, Stumpf PH, Harke H. Evidence for an apartheid-like social structure in early Anglo-Saxon England. Proc Roy Soc B. 2006;273: 2651-2657.

39) Bataille C, von Holstein ICC, Laffoon JE, Wilmes M, Liu XM, Davies GR. A bioavailable strontium isoscape for Western Europe: a machine learning approach. PLOS One. 2018;13: e0197386.

40) Collard M, Wood B. Homoplasy and the early hominid masticatory system: inferences from analyses of extant hominoids and papionins. J Hum Evol. 2001;41: 167-194.

Figure captions

Figure 1. Map depicting the locations of the five Anglo-Saxon cemeteries sampled in this study (red dots). The map also shows the homelands and destinations of the Germanic-speaking groups who settled in Britain between the mid 5th and early 7th centuries CE, the Angles (pink ellipses), Saxons (green ellipses), Jutes (purple ellipses), and Frisians (blue).

Figure 2. Location of 34 landmarks for capturing the shape of the basicrania.

Figure 3. Plots of CV values for female individuals.

Figure 4. Plots of CV values for male individuals.

Figure 1

Click here to access/download;Figure;Figure 1.jpg

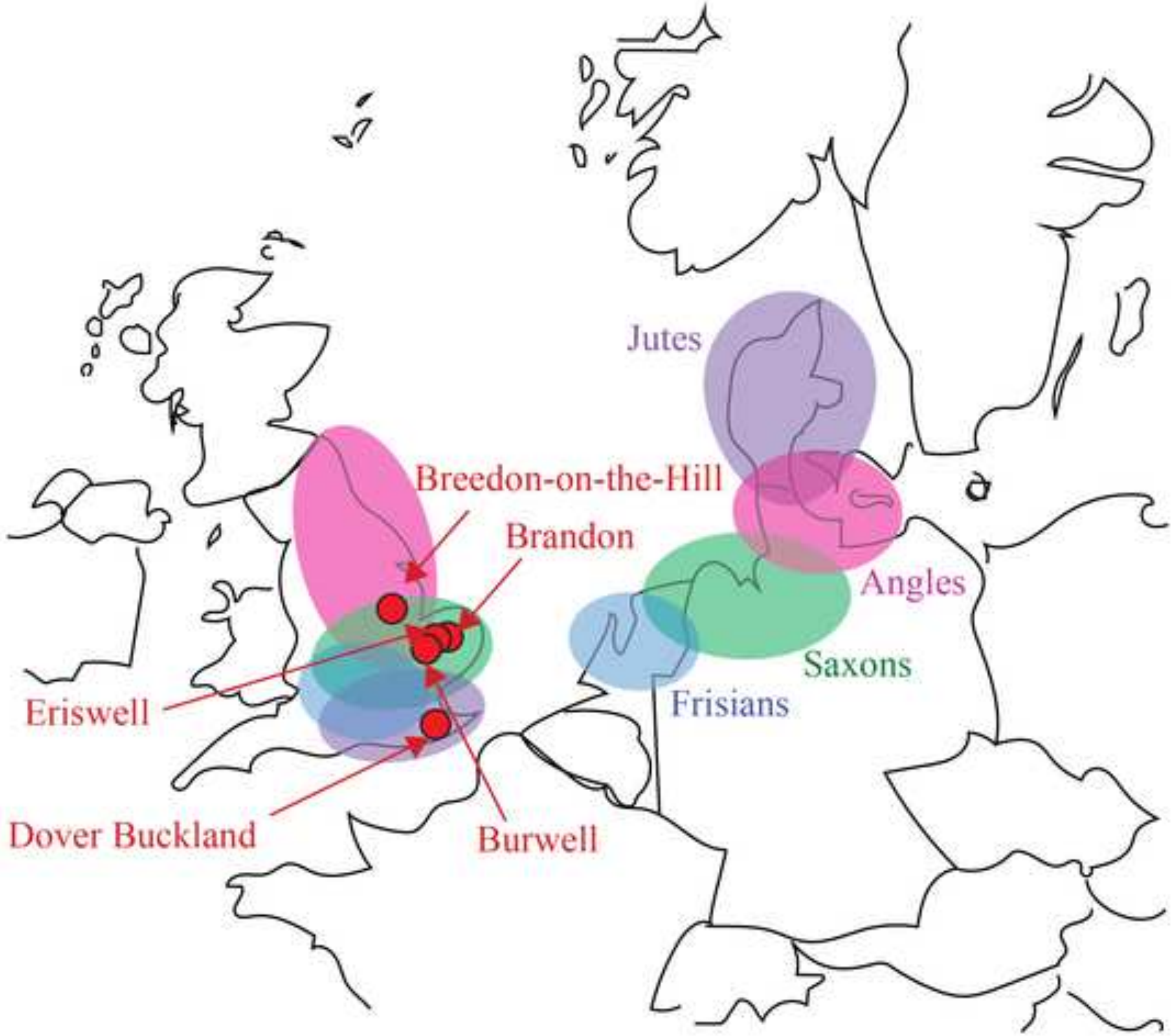

Click here to access/download;Figure;Figure 2.jpg

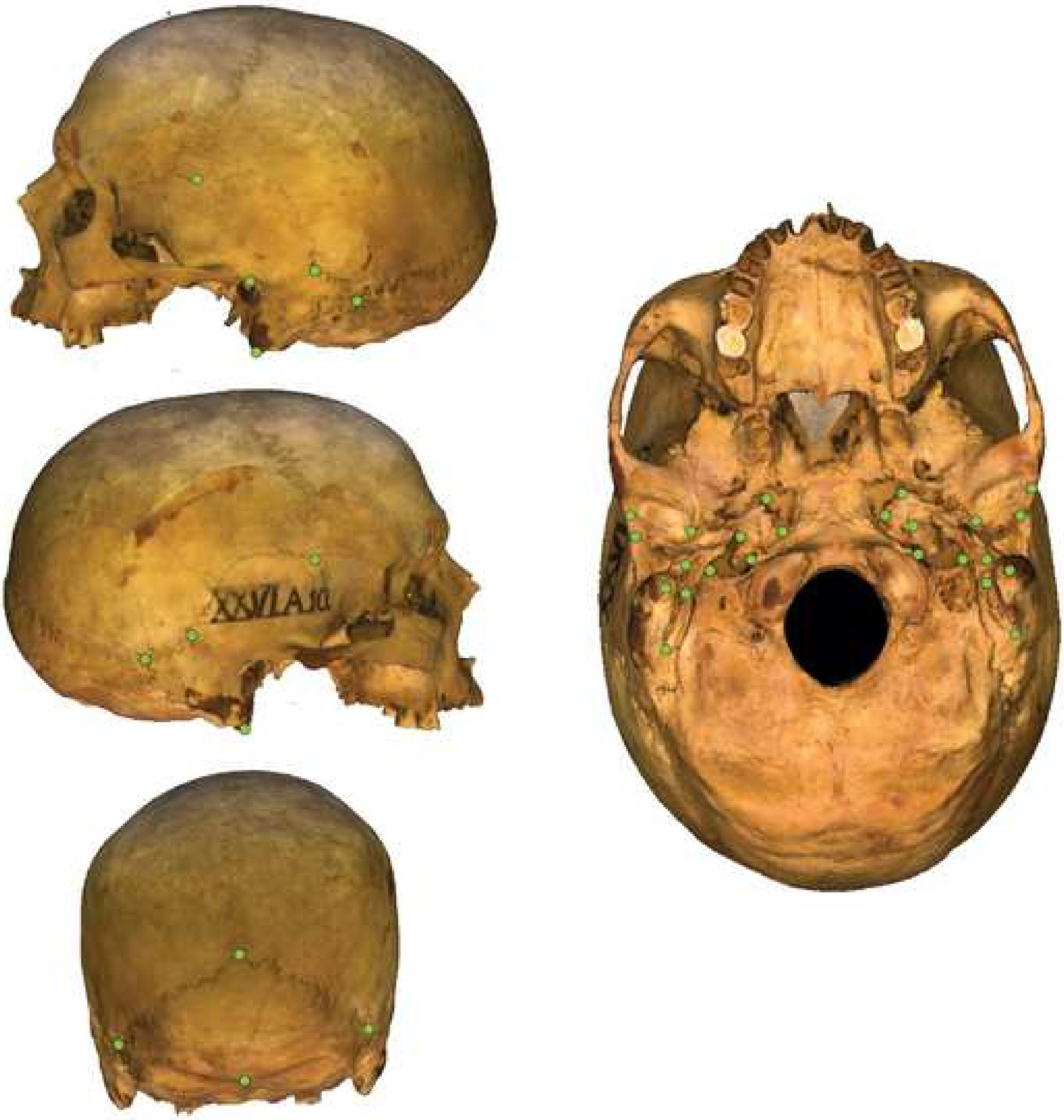

Figure 3

Click here to access/download;Figure;Figure 3.jpg

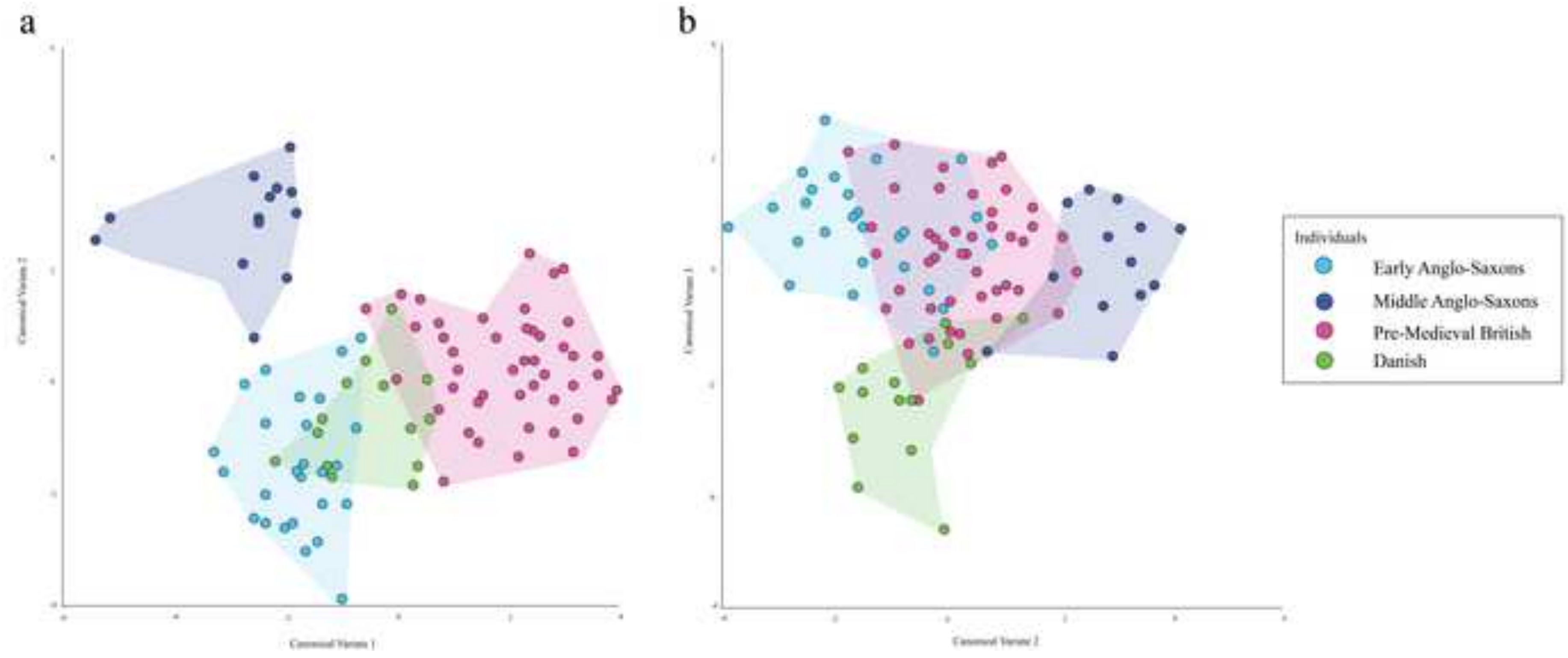

Figure 4

Click here to access/download;Figure;Figure 4.jpg

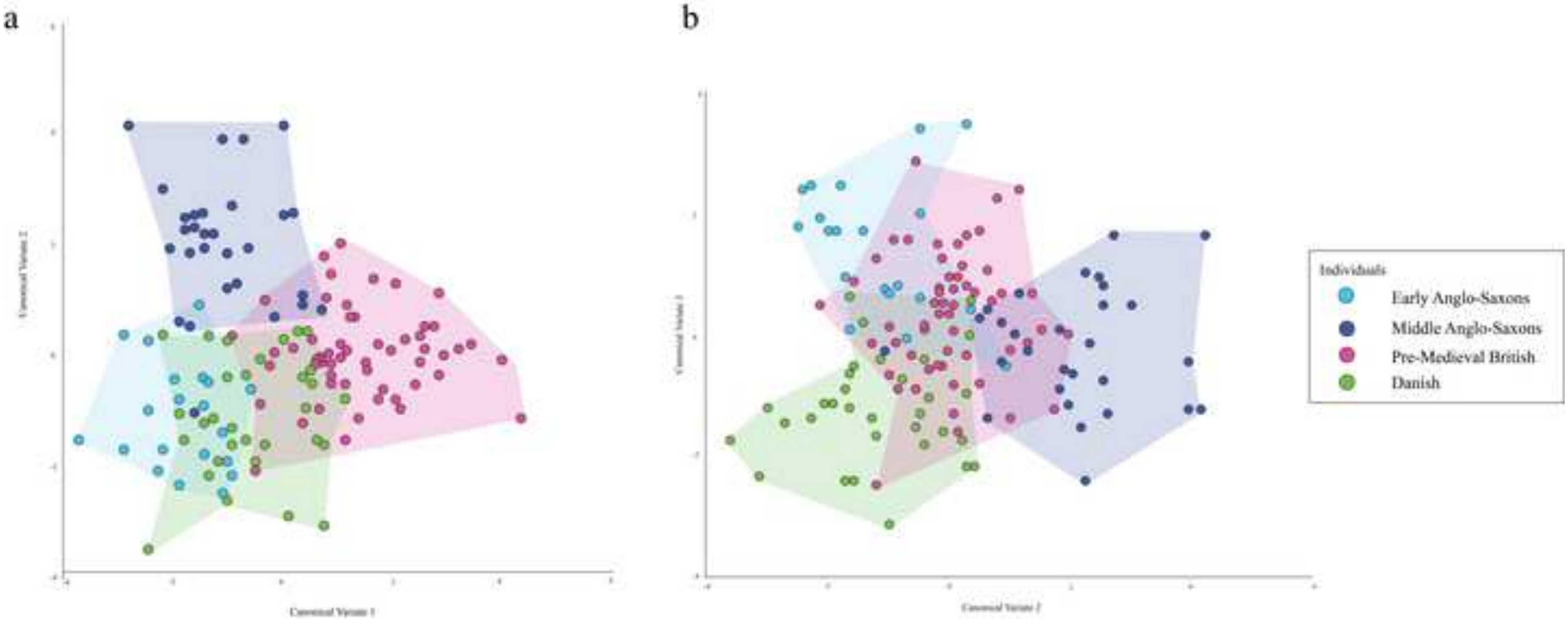

Click here to access/download
Supporting Information
Supplementary document.docx

Supporting Data

Click here to access/download
Supporting Information
Supplementary Data.txt
