# Supplementary material for: A 3D basicranial shape-based assessment of local and continental northwest European ancestry among 5th to 9th century CE Anglo-Saxons
Source: PLoS One. 2021 Jun 23;16(6):e0252477. doi: 10.1371/journal.pone.0252477 (PMC8221467; doi:10.1371/journal.pone.0252477)
Supplement: Attachment — Submitted filename: Responses to reviewers.docx [file pone.0252477.s005.docx]

**Responses to reviewers’ comments on the revised Plomp et al.’s ‘A 3D basicranial shape-based assessment of local and continental northwest European ancestry among 5^th^ to 9^th^ century CE Anglo-Saxons’**

Our responses to reviewers’ comments are below. Reviewer comments are in black text and our responses are in blue.

**Reviewer #1: I appreciate the effort the authors made in clarifying the methodological questions I had, as well as the addition of the new analyses. They have successfully addressed all my concerns, and this article will be a meaningful addition to the literature. My only remaining suggestions is to move the discussion of the supplementary analyses to the Results section, instead of the Discussion. I know that one of the other reviewers asked for this and the authors decided not to make these changes, but I'll echo that reviewer's opinion that it does not seems to fit well in the discussion, as it is not truly contributing to the contextualization of the data analyzed.**

Response: Thank you for the suggestion. We tried moving the supplementary analyses to the Results section, but we found that it did not work as well there as it does in the Discussion. It is also worth noting that the present structure matches that of paper we published in PLOS ONE earlier this year. So, there is precedent for including supplementary analyses in the Discussion in PLOS ONE papers.

**Reviewer #2: The authors sufficiently incorporated the more substantial issues brought up in the peer review; however, I am unsure if they saw or responded to the PDF'd document I originally uploaded with the first review. I don't see responses to some of the points brought up therein. In particular, I suggest changing "colonization" in the keywords to "ancestry" since the paper doesn't actually discuss colonization, but instead ancestry and population heterogeneity (ln 31); change "men" to "males" since we can't comment on gender as skeletal biologists, and the two terms shouldn't be conflated (ln 62); change "determined" to "estimated" when discussing sex since as skeletal biologists, we cannot determine the sex of someone--we can only estimate their assigned sex (ln 90). There are other minor comments in the PDF that can help with phrasing and clarification.**

Response: We apologise for this oversight. We had missed that there was a PDF with the first round of reviews. We thank the reviewer for taking the time to read the paper so thoroughly. We have edited the text in line with the suggestions in the PDF, with some minor modifications.

**Reviewer #3: I have noticed one remaining typo - In line 167, the word "difference" should be used rather than "different."**

Response: Well spotted. We’ve made the change.

**Reviewer #5: My original comment: "The authors repeated the LDA on a pooled sample to ascertain that the results from the sex-specific analyses are not biased. However, the pooled middle Anglo-Saxon sample remains predominantly male, so some uncertainty remains, as does the possibility of differential ancestry in males and females. Considering that some genetic data suggest high male continental ancestry, it would be worth pursuing this question, though I reckon this would require additional samples from Middle Anglo-Saxon females."**

**The authors' response:**

**"We agree that further research with a larger sample size would be beneficial, especially if combined with aDNA and isotope data. We hope to pursue this further in the coming years. As for the current analyses, the CVP PCs were calculated based on balanced samples, which should minimize any error due to sample size differences. This has now been added to Line 159"**

**This is not obvious to me: As clearly summarized in the discussion, the pooled LDA was carried out on the entire sample, not on a sex-balanced subsample, so my point remains unaddressed, or something needs to be clarified here. Note the acronym CVP is nowhere defined, I can only guess it refers to the pooled LDA. The reference to Line 159 seems erroneous.**

**In light of this, I still think that subsample sizes for female Danish and Middle Anglo-Saxons and male early Anglo-Saxons are small relative to the number of variables (PCs retained) and induce uncertainty for the classification results. I am not saying this uncertainty invalidates the interpretation, but it should be acknowledged in the discussion.”**

Sorry, we should have defined ‘CVP’ in the Materials and Methods. We have now replaced the acronym with ‘the PC-reduction procedure’, which hopefully is clearer.

The number of CVP PCs included in the main analyses did not exceed the smallest group size in each analysis, making them appropriate to use.

We don’t think that the results of our study support the male-bias hypothesis, because the LDA percentages for males and females are pretty similar (Table 3). Nevertheless, we have added some text to the ‘future directions’ part of the Discussion to suggest that potential sex differences in migration and acculturation should be examined with a more comprehensive sample. The relevant paragraph now reads as follows:

“There are two other obvious possibilities for future research. One is to repeat the study with a more comprehensive sample. In particular, it would also be useful to include individuals from regions of Britain not represented in the present study (see Figure 1). This would allow the hypothesis that there was regional variation in the relative number of Anglo-Saxons of continental northwest European and local ancestry to be evaluated. A more comprehensive sample would also allow the possibility that there were sex differences in migration and acculturation to be investigated. In the present study, the results for males and females were similar. However, other studies have suggested that the majority of incomers were male [2].”
